# The Safety Profile of Aspartame: A Review of Regulatory Standards and Emerging Health Concerns

**DOI:** 10.1155/jnme/4727546

**Published:** 2026-04-21

**Authors:** Hasan Uğur Öncel, Zehra Sökmen

**Affiliations:** ^1^ Nutrition and Dietetics Department, Health Sciences Faculty, Istanbul Gedik University, Istanbul, Türkiye

**Keywords:** aspartame, artificial sweeteners, cancer risk, human health, metabolic disorders, nervous system, phenylalanine

## Abstract

We reviewed the effects of aspartame, one of the artificial sweeteners widely used in the food industry, on human health. We evaluated the chemical structure, metabolic processes, and potential health effects of aspartame in light of scientific studies. Studies in various health areas, such as cancer risk assessment, effects on the nervous system and cardiovascular system, and its role in metabolic disorders, have led to various debates about the safety of aspartame for human health. While aspartame’s low‐calorie structure provides an advantage for metabolic diseases such as obesity and diabetes, its potential effects on insulin sensitivity and gut microbiota have led to different conclusions. In light of the adverse effects of the phenylalanine amino acid in the structure of aspartame on brain development, we have tried to emphasize that consumption levels should be more closely monitored in special risk groups such as phenylketonuria (PKU) patients, children, pregnant women, and the elderly. The widespread use of aspartame in the food industry has led to the view that this structure is safe when consumed as a sweetener at acceptable daily intake levels. This review seeks to answer, “Is it a safe additive for human health when individual sensitivities and long‐term effects are considered?”.

## 1. Introduction

Aspartame, an additive, has been widely used in food technology and artificial sweeteners. When its effects on human health have been evaluated in recent years, it has become a subject of broad debate in the academic field. In this review, starting from the discovery of aspartame, its regulatory approval processes, its widespread use in the food industry, consumption habits, and legal regulations were evaluated, and their effects on human health were discussed. Published academic papers and numerous experimental studies have demonstrated the potential benefits and possible risks of aspartame. The results have generated controversy among the scientific community and regulatory agencies [1]. In particular, the toxicological and biological evaluations that were initially put forward were observed even in model studies on the long‐term effects of aspartame. The findings obtained in systems such as *C. elegans* pointed to the product’s complex biological effects [2]. Our review aims to address the historical development systematically, as well as the uses and effects of aspartame in the light of available scientific data, and to provide guiding recommendations for future studies.

## 2. Scope and Uses of Aspartame

### 2.1. Discovery and Development of Aspartame

Aspartame is considered an important milestone in the development of modern artificial sweeteners. First discovered in 1965 by American chemist James M. Schlatter, it was discovered by chance during pharmaceutical research. At the same time, working on a stomach ulcer drug based on peptide synthesis, Schlatter accidently smeared the chemical compound he obtained on his finger, and felt a sweet flavor difference when he brought his finger to his mouth [2]. This event first revealed the potential of aspartame as a sweetener and aroused great interest in the scientific world.

The discovery of aspartame attracted attention not only because of its sweetness but also with its chemical structure. When its molecular structure was examined, it was found to be a dipeptide composed of phenylalanine and aspartic acid. This paved the way for new research on how aspartame can be metabolized in biological systems [3]. The fact that it gives a taste about 200 times higher than sugar in terms of sweetness has made it an effective artificial sweetener, even in low amounts. This property has made it important to develop alternative sweeteners for low‐calorie diets and diabetic patients [4].

Schlatter’s discovery attracted intense interest in the scientific world and was carefully followed by major companies in the food industry. Initial laboratory tests showed that aspartame exhibited a taste profile close to natural sugar but had an extremely low caloric value [5]. In particular, it has achieved a unique position among artificial sweeteners due to its chemical stability and contribution to food flavor profiles.

The transition of aspartame from pharmaceutical research to the food industry has sparked considerable debate in the scientific community. Early research involved various biochemical assays to assess the potential effects of aspartame on human health. In this context, it has been determined that aspartame consumption may carry a risk in individuals with genetic diseases related to phenylalanine metabolism (e.g., phenylketonuria (PKU) patients) [6]. However, studies for the general population have determined that it is safe when consumed within the specified daily intake amounts [7]. In the early 1970s, G. D. Searle & Company filed the first patent applications for using aspartame as a sweetener. In this process, extensive safety tests and clinical studies, which had to be approved by regulatory authorities, were conducted to bring the product to the market [8]. Studies have shown that aspartame is stable in terms of taste profile and can maintain its taste characteristics to a great extent, even under different temperature conditions.

However, one of the biggest challenges in commercializing aspartame has been to answer the safety concerns of decision‐making authorities. While some of the toxicological studies in the first phase suggested that it may have carcinogenic effects when consumed in high doses [9], later comprehensive analyses showed that it does not pose any health risk within the safe consumption limits set. Therefore, approval by the US Food and Drug Administration (FDA) was only possible after intense scientific debate [10].

The cost advantage of aspartame compared to traditional sucrose (tea sugar) has led to its use in the food industry. Offering it as an alternative to sucrose from sugar beet and sugar cane has provided a significant cost advantage, especially for companies producing low‐calorie food products [11]. The raw material supply and production processes required for the industrial production of aspartame are considered more sustainable than that of other artificial sweeteners, leading to its widespread adoption in the international market [12].

The acceptance of aspartame as a sweetener in the food industry is directly linked to health policies and dietary practices, especially those aiming to reduce sugar consumption.

Toward the end of the 1980s, with the increase in obesity and diabetes worldwide, the popularity of aspartame‐containing products rose rapidly [13]. Its use has become widespread in a wide range of products, from carbonated drinks to yogurts and chewing gum to sweetener tablets. It has been used in significant products such as carbonated cola drinks worldwide [13].

Beverage brands, which have a market, started using aspartame as an essential sweetener in diet products and introduced different products to the market [14].

The marketing process of aspartame is based on consumer perception and scientific data. Advertising campaigns emphasized the advantages of aspartame as a low‐calorie and healthy alternative, leading to a significant shift in consumer preferences. However, some studies have suggested that long‐term consumption of aspartame may have neurological effects, leading to continued controversy [15]. Therefore, safety assessments by food inspection agencies continue to monitor the public health impacts of aspartame continuously.

### 2.2. Regulatory Approval Process

The regulatory approval process for aspartame is a multistage process that has set global precedents for safety assessments of food additives. The US FDA conducted extensive scientific reviews and long‐term toxicological studies to determine the safety of aspartame in food products. First, in 1974, the FDA approved using aspartame as a tabletop sweetener. However, this approval was suspended due to potential health risks raised by some at that time, and more extensive research was requested [16].

These studies on aspartame safety covered various issues, including genotoxicity, carcinogenicity, neurotoxicity, and metabolic effects. In 1981, following further investigations, the FDA reapproved aspartame for use in dry foods. This decision was based on data provided by G. D. Searle & Co. and the results of independent studies [17].

In 1983, it was approved for use in carbonated beverages, and aspartame has since become a widely consumed sweetener worldwide [18]. One of the most important factors taken into the FDA’s 117 account during the decision process concerns the metabolic process of aspartame.

Aspartame is broken down into components such as phenylalanine, aspartic acid, and methanol in the body. The controversy about the toxic effects of methanol has forced regulatory agencies to take a more careful look [10]. However, studies have shown that methanol intake from aspartame is significantly lower than that of fruits and vegetables [20].

The FDA has also set an acceptable daily intake (ADI). This value represents the maximum amount of aspartame that individuals can safely consume daily for a lifetime. [20]. This value includes a wide margin of safety to ensure the safety of consumers. The FDA’s decision was based on toxicological and clinical studies in which aspartame was tested at different doses.

The FDA’s evaluation process is not limited to scientific data but includes public hearings organized to protect public health. In public hearings organized in the mid‐1980s, scientists, health experts, and members of the public came together to discuss the safety of aspartame [21]. This process was part of the FDA’s transparency‐based scientific and social evaluation mechanism.

#### 2.2.1. World Health Organization (WHO) and Codex Alimentarius Standards

The international acceptance of aspartame is based on safety standards set by the WHO and the Codex Alimentarius Commission. The Codex Alimentarius Commission, established in collaboration with the WHO and the Food and The Agriculture Organization of the United Nations (FAO), sets international standards for food additives to protect consumer health and promote fair trade practices [22].

Codex Alimentarius has conducted safety assessments on using aspartame in foods through the Joint Committee of Experts on Food Additives (JECFA). JECFA has reviewed many studies on the toxicological profile, metabolism processes, potential carcinogenic effects, and effects of aspartame. As a result of these evaluations, it was concluded that aspartame is safe within a specific dose range. The ADI value set by Codex is a specific dose range [23]. The ADI value set by Codex Alimentarius is very similar to that set by the FDA. WHO and FAO set the ADI for aspartame at 40 mg/kg body weight [24]. This value indicates that the daily dietary intake of aspartame is at a level that does not negatively impact health. In addition, this value was determined by considering different age groups, genders, and health conditions. The standards set by Codex Alimentarius are used as a reference in the food safety regulations of many countries around the world. Especially in developing countries, regulations on food additives are based on Codex standards to ensure that international trade is carried out [25].

#### 2.2.2. European Food Safety Authority (EFSA) Assessments

The EFSA is the main body assessing the safety of aspartame in the European Union (EU). EFSA’s assessments of aspartame are based on new findings in the scientific literature and toxicological studies. EFSA conducted comprehensive risk assessments on the safety of aspartame in 2009 and 2013 [26]. Published in 2013, EFSA’s scientific opinion found that aspartame is not genotoxic, carcinogenic, or neurotoxic and can be safely consumed within the specified ADI values. EFSA has set the ADI for aspartame at 40 mg/kg body weight. This value is based on long‐term consumption and possible chronic effects [27]. Another important issue that EFSA considers during the evaluation process is the metabolism processes of aspartame.

In the digestive system, aspartame is broken down into phenylalanine, aspartic acid, and methanol: The effects of these components on the body are evaluated separately. Since phenylalanine intake may pose a risk, especially for patients with PKU, special warnings have been issued for these individuals [13]. EFSA worked with an extensive scientific advisory board of public health experts, toxicologists, neurologists, and epidemiologists during the evaluation process. This advisory board meticulously examined the potential health risks of aspartame and determined that aspartame can be used as a safe additive in foods [28].

### 2.3. Uses of Aspartame in the Food Industry

#### 2.3.1. Diet Drinks, Sweeteners, Dairy Products, Gum, and Confectionery

Aspartame is one of the most widely used compounds among low‐calorie sweeteners. The use of aspartame in the food industry is seen in a wide range of products, particularly diet drinks, tabletop sweeteners, dairy products, chewing gum, and confectionery.

The fact that aspartame is about 200 times more flavoring than sugar allows it to provide effective sweetening even in tiny amounts, and this feature is a preferred factor, especially for individuals who want to reduce their energy intake [16].

Diet drinks are among the products where aspartame is most widely used. Cola and similar soft drinks use aspartame as the primary sweetener in their low‐calorie beverages. The transparent taste profile of aspartame and its compatibility with other ingredients help to maintain taste balance in these beverages [17]. Aspartame is unstable to prolonged heating and becomes tasteless upon breakdown. Therefore, it is not suitable for baking and cooking. Because of its temperature sensitivity, aspartame generally performs better in cold beverages. Tabletop sweeteners are another essential use of aspartame. Brands such as Equal and NutraSweet prefer aspartame in their products, which are used to sweeten coffee, tea, and other beverages [18]. These products are especially popular among diabetic patients and individuals on low‐carbohydrate diets. Aspartame is also widely used in dairy products. Low‐calorie yogurts, flavored milk, and desserts are the main products in which aspartame is used.

Aspartame replaces sugar in these products, reducing calories that help maintain the sweetness profile [10]. Chewing gum and candies are also among the product categories, where aspartame is used extensively. Sugar‐free chewing gums and diet candies contain aspartame to help prevent dental caries. These products are beautiful for individuals sensitive to oral health [19].

#### 2.3.2. Role in Dietary Supplements

Aspartame is widely used in food products, pharmaceuticals, and dietary supplements. It is preferred as a sweetener in the pharmaceutical industry, especially in chewable tablets. Lozenges and syrups are produced for children and the elderly [20]. While aspartame’s taste profile masks bitter or sour taste, it does not affect the efficacy of drugs. Aspartame also plays an important role in nutritional supplements and sports products. Aspartame provides a sweet flavor without increasing calories in products such as protein powders, energy bars, and electrolyte drinks. This is a great advantage for athletes and individuals who want to control weight [21]. The use of aspartame in drugs is generally aimed at increasing patient compliance with treatment. Masking unpleasant tastes allows pediatric and geriatric patients to consume drugs more easily [22]. In addition, it facilitates dosage control by providing high sweetness at low doses and reducing costs. In this respect, using aspartame in the food industry is a sweetener and part of a multifaceted strategy to address health, economics, and consumer preferences. Aspartame has an important place in the food, pharmaceutical, and dietary supplement industries, thanks to its advantages across a wide range of products and safety approvals from regulatory agencies [18, 29–31].

#### 2.3.3. Aspartame‐Containing Products and Consumption Habits

Aspartame has become an important component of the food industry worldwide and has found a place in the product portfolio of many international brands.

Equal and NutraSweet have developed products that appeal to a wide range of consumers by taking advantage of the low‐calorie sweetening properties of aspartame [1, 8]. Diet drinks, such as soft drinks containing Cola, are among the products in which aspartame is widely used. In these products, aspartame replaces sugar and provides sweetness with fewer calories, thus offering an attractive alternative to consumers regarding weight control and diabetes management. Tabletop sweetener brands such as NutraSweet and Equal are formulated specifically for coffee, tea, and cold drinks. Beyond providing sweetness, these products are an important alternative for individuals who want to reduce sugar consumption [3, 6]. These products, which are sold as sweetener tablets or powders, provide the practical ease of use in daily life. Furthermore, the application of aspartame in the pharmaceutical sector is quite common. It is preferred to mask the bitter taste, especially in pediatric drugs, chewable tablets, and syrups [21, 22].

Aspartame is also widely used in many processed foods, such as frozen desserts, yogurts, chewing gums, and energy drinks. The main reason why aspartame is preferred in these products is that it has high sweetness intensity and can provide the desired sweetness in tiny amounts [4, 9]. This feature reduces production costs and, at the same time, minimizes the calories of the products. For example, in sugar‐free chewing gums, aspartame is used instead of simple sugars such as glucose and fructose, which can cause dental caries and provide a feature that supports dental health [19].

Aspartame consumption varies worldwide. Various factors influence it, such as cultural dietary habits, health policies, consumer awareness, and economic conditions. The high demand for dietary products in North America and Western Europe has led to widespread aspartame consumption. In the USA and Canada, high rates of obesity and diabetes have directed consumers to low‐calorie sweeteners instead of sugar [10, 13].

Studies conducted in the USA reveal that many individuals regularly consume aspartame‐containing products in their daily diets [10].

Aspartame consumption has been limited in regions such as Asia and the Middle East due to the predominance of traditional dietary habits. For example, in countries such as Japan, the natural sweeteners stevia and monk fruit are more popular.

However, with the effect of globalization, diet drinks and processed foods containing aspartame consumption have increased in these regions [5, 12]. In Latin America, especially in countries such as Brazil and Mexico, aspartame consumption has increased rapidly within the scope of increasing diabetes rates and policies to combat obesity. Local regulations have encouraged the taxation of sugary drinks while allowing low‐calorie alternatives to find more space in the market [15, 23]. Similarly, aspartame is supported in European Union countries within the framework of healthy nutrition policies.

Safety assessments conducted by EFSA have helped European consumers make more informed decisions about aspartame [26]. In Africa, aspartame consumption is lower than in other regions. This relates to the economy factors, low‐income levels, and limited market access. However, with the modernization and adoption of Western lifestyles in large cities, consumption of aspartame‐containing products is increasing [13, 18].

#### 2.3.4. Legal Regulations and Consumption Limits

International Health sets ADI levels for the safe consumption of aspartame to protect public health. The ADI is the maximum amount of aspartame that an individual can consume regularly every day throughout their lifetime. The FDA has set the ADI for aspartame at 50 mg/kg body weight [20]. This value ensures that health risks are kept to a minimum, even in the case of excessive consumption. The EFSA has adopted a more cautious approach to ADI for aspartame, which was determined to be 40 mg/kg [27]. This difference is due to the different risk assessment methods used by the regulatory agencies. EFSA’s assessments take into account the protection of vulnerable groups such as children, pregnant women, and people with chronic diseases. When setting ADI values, data from animal studies and human clinical trials are considered. Especially, toxicological studies are important for the assessment of the potential health risks of consuming aspartame in high doses [7, 14]. Studies show that aspartame consumption within ADI limits does not cause serious health problems such as cancer, neurological diseases, or metabolic disorders [9, 25].

#### 2.3.5. Legal Regulations in Different Countries

The legal status of aspartame varies around the world. Many countries have laws and regulations governing the use of aspartame in food products that have been developed.

The FDA has confirmed that aspartame is safe and has authorized its use in a wide range of foods [17]. Products containing aspartame must include warnings, especially for patients with PKU. This is also important for individuals with phenylalanine sensitivity.

In the European Union, aspartame is used by regulations set by the European Parliament and Council. The regulations on using aspartame as a food additive are based on the scientific opinions of EFSA [26]. In EU countries, it is mandatory to include the statement “source of phenylalanine” on the labels of products containing aspartame. Countries such as Japan, South Korea, and Australia also allow the use of aspartame. In Japan, aspartame has been used in various food products since the 1980s. The Japanese Ministry of Health, Labor, and Welfare (MHLW) has stated that aspartame is safe and regularly conducts safety assessments [4, 19].

Aspartame is also widely used in Latin American countries such as Brazil, Mexico, and Argentina. Aspartame has become more prevalent in these countries due to taxation policies on sugary drinks [15, 24]. Especially in Mexico, additional taxes were introduced for sugary drinks as part of the fight against obesity, which increased interest in low‐calorie sweeteners such as aspartame. Aspartame is regulated under international trade agreements in African countries such as South Africa, Nigeria, and Kenya. In these countries, Codex Alimentarius standards are generally taken as a basis [18, 29–31].

## 3. Metabolism of Aspartame

### 3.1. Chemical Structure and Physicochemical Properties

Aspartame is a compound with the chemical formula C_14_H_18_N_2_O_5_. classified as an artificial sweetener. This molecule comprises two essential amino acids—L‐aspartic acid and L‐phenylalanine—joined by a dipeptide ester bond. The phenylalanine part of this structure is esterified with methanol, giving the molecule a structure that enhances its sweetness properties. The molecule is partially soluble in water due to the presence of the ester bond, but it is sensitive to temperature, and its flavor profile deteriorates at high temperatures [1, 4].

Since its degree of sweetness is about 200 times higher than that of sucrose, it is possible to achieve the desired level of sweetness by using tiny amounts. This feature helps maintain the desired level of sweetness by using tiny amounts. This feature helps maintain the feeling of sweetness while keeping the calorie amount to a minimum, especially in diet products [2, 7]. The phenylalanine component in the molecular structure creates intense sweetness by interacting strongly with taste receptors thanks to its aromatic ring structure. The isomeric structure of aspartame is also important. Its presence in the form of L‐aspartic acid and L‐phenylalanine makes it compatible with biological systems. However, toxic effects occur in the presence of D‐isomers, so stereochemical purity is meticulously maintained during industrial production [9]. The pH stability of the molecule is limited, especially in acidic or high‐temperature environments; it undergoes hydrolysis and loses its sweetness. Therefore, pH balance is critical in carbonated beverages [12]. Aspartame is a white crystalline, odorless, and slightly sweet powder when its physicochemical properties are examined. The melting point is 246–247°C, and it has no hygroscopic properties. Its solubility in water is approximately 10 mg/mL, and the degree of solubility increases in direct proportion to temperature. Its solubility in organic solvents is relatively low [13].

### 3.2. Components of Aspartame

Aspartame is mainly composed of three components: L‐aspartic acid, L‐phenylalanine, and methanol. These components are released when aspartame is broken down in the body and take on different functions in biological systems.

#### 3.2.1. L‐Aspartic Acid

L‐aspartic acid is one of the important components of aspartame. It is naturally present in many protein structures. This compound stands out among amino acids with its polar structure and is important in protein synthesis, the urea cycle, and energy metabolism. [5]. Aspartic acid acts as a neurotransmitter in the brain. It acts as an excitatory neurotransmitter in the synaptic transmission, especially when working with glutamate. However, it is thought to cause neurotoxic effects when taken in excessive amounts, leading to a condition called “excitotoxicity” [10].

#### 3.2.2. L‐Phenylalanine

L‐phenylalanine, another component of aspartame, is an essential amino acid and a compound that the body must take from outside for protein synthesis. Phenylalanine is converted to tyrosine and produces neurotransmitters such as dopamine, norepinephrine, and epinephrine [6, 11]. However, phenylalanine cannot be metabolized in individuals with PKU and reaches toxic levels. This leads to severe neurological damage. For this reason, products containing aspartame must include warnings for PKU patients, such as “contains phenylalanine” [15].

#### 3.2.3. Methanol

Methanol, which is released during the metabolism of aspartame, is one of the most controversial ingredients due to its toxicity potential. Methanol is metabolized to formaldehyde and then to formic acid in the body. This conversion process causes toxic effects at high doses [8, 14]. However, methanol from aspartame is lower than that found naturally in many fruits and vegetables. For example, methanol in a glass of tomato juice is higher than in a diet drink containing aspartame [3, 10].

### 3.3. Metabolism Process and Breakdown in the Body

When taken orally, aspartame is rapidly broken down in the gastrointestinal tract and decomposed into its components: L‐aspartic acid, L‐phenylalanine, and methanol. This process occurs through enzymatic hydrolysis and begins with aspartame absorption in the intestines [4, 9]. Enzymes in the stomach and small intestine hydrolyze aspartame. In this process, peptidase enzymes are activated, and the dipeptide bond of aspartame dissolves. Aspartic acid and phenylalanine are absorbed from intestinal epithelial cells and pass into the bloodstream [2, 13]. Methanol is rapidly absorbed in the intestine and transported to the liver. In this process, there is no sensation of sweetness because aspartame’s sweetness depends on its molecular integrity before it is broken down. In the liver, methanol is oxidized to formaldehyde and formic acid.

During this conversion, formaldehyde is present in the circulation for a short time and is rapidly converted to formic acid and metabolized to carbon dioxide. Formic acid is detoxified through mitochondrial metabolism and excreted through urine [12, 20]. L‐phenylalanine is converted to tyrosine in the liver and used for neurotransmitter synthesis. On the other hand, L‐aspartic acid contributes to energy production by entering the Krebs cycle [7, 15].

### 3.4. Biological Role and Toxicological Effects of Metabolites

Aspartame’s components, phenylalanine, aspartic acid, and methanol, have different biological functions in the body. However, some toxic risks arise in the case of excessive consumption. While phenylalanine assumes important biological functions in normal individuals, it causes toxic effects that inhibit brain development in PKU patients. PKU patients are unable to metabolize phenylalanine due to the phenylalanine hydroxylase enzyme deficiency. This causes high levels of phenylalanine that can cross the blood–brain barrier, leading to mental retardation, seizures, and behavioral disorders [11, 16]. Therefore, PKU warnings are mandatory on products containing aspartame. Aspartic acid acts as an excitatory neurotransmitter in the central nervous system. It increases neuronal excitability, especially by working together with glutamate. However, excessive aspartic acid intake leads to excitotoxicity causing neurologic impairments [6, 19]. Excitotoxicity occurs when patients cannot metabolize phenylalanine due to phenylalanine hydroxylase enzyme deficiency. This causes elevated phenylalanine levels that can cross the blood–brain barrier, leading to mental retardation, seizures, and behavioral disorders [11, 16].

Therefore, PKU warnings are mandatory on products containing aspartame. Aspartic acid acts as an excitatory neurotransmitter in the central nervous system. It increases neuronal excitability, especially when working together with glutamate. However, excessive aspartic acid intake leads to excitotoxicity and causes neurologic impairments [6, 19]. Excitotoxicity has been associated with neurological diseases such as Alzheimer’s disease and epilepsy. Methanol toxicity is one of the most controversial issues related to the safety of aspartame. The conversion of methanol to formaldehyde and formic acid causes toxic effects, especially at high doses. However, the amount of methanol from aspartame is well below toxic doses. For example, the amount of methanol from 1 L of the diet drink is lower than that from naturally occurring fruit juices [8, 14, 21].

### 3.5. Energy Conversion and Caloric Value of Aspartame

Aspartame is a low‐calorie sweetener. It provides approximately 4 kcal of energy per gram, but since it is used in tiny amounts, its total caloric contribution is negligible [1, 10]. The low‐calorie nature of aspartame is ideal for individuals who want to achieve weight control. When used as a sugar substitute, it significantly reduces energy intake. This is an important factor in reducing the risk of obesity, diabetes, and cardiovascular disease [5, 10]. Aspartame does not affect blood sugar levels because its glycemic index is nearly zero. This feature is essential for patients with type 2 diabetes. Studies have shown that aspartame does not trigger an insulin response and does not negatively affect glycemic control [3, 9, 17].

## 4. Effects of Aspartame

### 4.1. Potential Health Risks

#### 4.1.1. Cancer Risk: Evidence on Brain Tumors and Leukemia

The potential carcinogenic effects of aspartame have been the focus of scientific research, especially since the 1990s. These discussions are based on the cancer risk findings observed in some animal studies and epidemiological data. The first studies linking aspartame to brain were done in 1996, where 422 tumors came to the fore through an epidemiological analysis. In this study, it was reported that an increase in the incidence of brain tumors was observed after aspartame became commercially available [1]. However, there has been considerable debate in the scientific community about whether this correlation is based on a cause‐and‐effect relationship. Long‐term animal studies conducted by the European Ramazzini Foundation in Italy have shown that high doses of aspartame may increase the risk of leukemia and lymphoma in some rat models [2]. In these studies, a significant increase in cancer incidence was observed, especially in rats exposed to aspartame during the prenatal period. However, the direct association of these results with human health has been debated because the doses of aspartame given to rats under experimental conditions are much higher than the regular consumption levels in humans [3].

Looking at human studies, large‐scale cohort studies and meta‐analyses show no consistent association between aspartame consumption and cancer risk. For example, a study of 285,000 people published in 2006 reported that aspartame consumption was not significantly associated with brain tumors, leukemia, or other types of cancer [4]. Similarly, a sizeable epidemiologic study conducted by the United States National Cancer Institute found no significant association between aspartame consumption and cancer incidence [5].

However, some studies have hypothesized that the toxic effects of methanol, one of the breakdown products of aspartame, may indirectly trigger cancer development. The conversion of methanol in aspartame to formaldehyde, which is considered a carcinogen, may make this sweetener eligible for carcinogen designation. Still, the amount of methanol from aspartame is reported to be much lower than the levels of methanol found naturally in many fruits and vegetables [6].

#### 4.1.2. Neurological Effects: Headache, Depression, and Seizure Risk

The neurological effects of aspartame have been evaluated in the context of various symptoms such as headache, depression, and seizure risk. Headache is one of the neurologic symptoms most commonly associated with aspartame consumption. Some case studies and reports in the 1980s described individuals who experienced headaches, particularly following the consumption of diet drinks [8]. These cases paved the way for larger‐scale clinical trials to investigate the potential effects of aspartame on the central nervous system. The results of randomized controlled trials are mixed. Some studies have shown that aspartame consumption may increase headache frequency in individuals with a history of migraine [9].

Other studies have not confirmed such an association [10]. The headache‐triggering potential of aspartame may be related to individual sensitivities and genetic differences. This is mainly explained through biochemical pathways affecting neurotransmitter balance. Aspartame breakdown products, such as phenylalanine and aspartic acid, affect neurotransmitter levels and trigger headaches in sensitive individuals [11].

Depression and mood disorders have also been examined among the potential neurological effects of aspartame. Some studies have suggested that a high‐dose aspartame consumption may increase depressive symptoms by affecting serotonin levels [12]. This is due to the potential for phenylalanine to block the passage of tryptophan, the precursor of serotonin, into the brain. However, this effect is usually associated with excessive consumption and is not evident at normal dietary intake levels. This is due to the potential for phenylalanine to block the passage of tryptophan, the precursor of serotonin, into the brain.

However, this effect is usually associated with excessive consumption and is not evident at normal dietary intake levels. A 2014 meta‐analysis reported no consistent association between aspartame consumption and depression in the general population [13].

Aspartame administration may lower the seizure threshold [14]. However, controlled clinical studies in humans have not confirmed these findings, and there is insufficient evidence that aspartame triggers epilepsy or other seizure disorders [15]. However, the neurologic effects of aspartame are more pronounced in individuals with specific genetic susceptibility, such as PKU patients.

#### 4.1.3. Effects on Cardiovascular Health

The effects of aspartame on cardiovascular health have also been an important topic of research. In particular, the possible effects of artificial sweeteners on metabolic syndrome, hypertension, dyslipidemia, and cardiovascular diseases are discussed. There is no substantial evidence that aspartame has direct toxic effects on the cardiovascular system; its indirect effects should not be ignored [16]. Many observational studies have reported a weak association between aspartame consumption and cardiometabolic diseases. For example, a large cohort study published in 2017 showed a slightly increased risk of stroke and heart attack in individuals who regularly consumed diet drinks [17].

However, it is difficult to conclusively establish a cause‐and‐effect relationship in such studies because individuals who consume aspartame tend to have metabolic risk factors regarding their living conditions and dietary tendencies.

The effects of aspartame on glucose and insulin metabolism have also been studied.

Although some studies have suggested that artificial sweeteners may negatively affect insulin sensitivity [10], aspartame has not significantly affected glycemic response. Studies in diabetic patients have confirmed that aspartame does not adversely affect blood glucose control [19].

At the mechanistic level, it has been hypothesized that methanol, one of the breakdown products of aspartame, may affect endothelial function through oxidative stress. Oxidative stress is one of the main pathophysiologic mechanisms of cardiovascular diseases, such as atherosclerosis [21]. However, methanol levels caused by aspartame consumption are far below the level that would cause such effects unless excessive consumption is directed.

In general, it is not easy to make a definitive judgment on the effects of aspartame on cardiovascular health. Current evidence suggests that aspartame consumed below ADI levels does not pose a cardiovascular risk [21]. However, long‐term prospective studies are needed to better assess potential risks in older age groups and individuals with chronic diseases.

#### 4.1.4. Scientific Debate on the Safety of Aspartame

The EFSA and the US FDA, among the most authoritative bodies on aspartame safety, have conducted comprehensive safety assessments examining the health effects of aspartame over the years.

As a result of its comprehensive review of the safety of aspartame in 2013, EFSA reported that aspartame is safe when consumed at specified ADI levels (40 mg/kg body weight) [22]. This assessment was based on a systematic review of hundreds of scientific studies covering the possible effects of aspartame on genotoxicity, carcinogenicity, neurotoxicity, and reproductive health. EDA approved aspartame for dry foods in 1981 and authorized its use in carbonated beverages in 1983. The FDA reviewed more than 100 toxicologic and clinical studies supporting the safety of aspartame and determined that the ADI level of aspartame is 50 mg/kg [23]. The FDA’s evaluations included animal studies, human clinical trials, and epidemiologic data. Both organizations evaluated the potentially toxic effects of phenylalanine, aspartic acid, and methanol, the metabolic byproducts of aspartame. These assessments emphasized that methanol from aspartame was lower than that from naturally consumed fruits and vegetables [24].

A significant part of the controversy about the safety of aspartame stems from differences between the results obtained from animal and human studies. Some studies in animal models have reported links between high‐dose aspartame exposure and cancer, neurological disorders, and metabolic dysfunctions [25]. For example, studies in rats have observed that high doses of aspartame may increase the incidence of leukemia and other diseases.

Therefore, results obtained in animal models should only be considered an indication of their potential impact on human health [13].

Human studies are often more complex because many variables, such as dietary habits, genetic predisposition, lifestyle factors, and environmental exposures, are involved.

Randomized controlled trials are better at assessing the short‐term effects of aspartame, but prospective cohort studies are needed to examine long‐term health effects [13].

#### 4.1.5. Effects of Aspartame on the Metabolic and Endocrine System

The effects of aspartame on obesity and weight management have been extensively studied, especially with the increasing use of artificial sweeteners over the past few decades.

When aspartame is used as a low‐calorie sweetener instead of sugar, it effectively manages weight by reducing the daily calorie intake. This has been considered an important advantage, especially for individuals who want to achieve weight control and for societies struggling with obesity [1, 3]. However, some studies suggest that aspartame may paradoxically contribute to weight gain. These claims are based on the hypothesis that aspartame may stimulate reward centers in the brain through the perception of sweetness, leading to increased appetite [4].

Some animal studies have shown that long‐term aspartame consumption may promote weight gain by affecting energy balance. For example, in a mouse study, an increase in insulin resistance and abdominal adiposity was observed with aspartame consumption [5]. However, the data from human studies are more complex. In randomized controlled trials, aspartame effectively reduces energy intake and body weight. Especially in individuals in low‐calorie diet programs, aspartame has been found to increase compliance with calorie restriction by meeting the need for sweets [6].

One of the mechanisms proposed to explain the effects of aspartame on obesity is the role of sweetness perception on metabolic responses. Sugary taste perception triggers insulin release.

However, aspartame creates a “metabolic illusion” in this process since it does not provide a caloric content. It has been suggested that this may adversely affect energy balance in the long term [7]. However, meta‐analysis studies do not provide consistent evidence that aspartame causes weight gain. On the contrary, it has been reported that low‐calorie sweeteners may be beneficial for weight loss or maintenance [8].

The effects of aspartame on insulin resistance and glucose metabolism are of great importance, especially for diabetes management. Studies show that aspartame does not directly affect blood glucose levels and is considered a sweetener with a glycemic index close to zero [9]. This feature has made aspartame a suitable alternative for diabetic patients.

However, some studies suggest that aspartame may indirectly affect insulin responses.

Some studies in animal models have shown that long‐term aspartame consumption may lead to insulin resistance. For example, in an experiment in rats, insulin sensitivity was decreased with high doses of aspartame consumption [10]. This effect may be related to aspartame altering the gut microbiota, significantly affecting glucose metabolism [11].

In human studies, the effects of aspartame on insulin resistance are more uncertain.

In randomized controlled trials, it was reported that aspartame consumption did not significantly change the insulin response in healthy individuals [12]. Similar results were obtained in studies conducted on diabetic patients. It has been shown that aspartame does not adversely affect blood glucose control or increase the risk of hyperglycemia [13].

#### 4.1.6. Neurological and Behavioral Effects of Aspartame

Aspartame’s effects on neurological functions have been linked to attention deficits and concentration problems, in particular. Phenylalanine, one of the breakdown products of aspartame, has a significant effect on the central nervous system. Phenylalanine is a precursor of neurotransmitters such as dopamine and norepinephrine, and these neurotransmitters play critical roles in cognitive functions, including attention, learning, and memory [15].

Some studies have shown that high‐dose aspartame consumption may worsen symptoms of attention‐deficit hyperactivity disorder (ADHD). This hypothesis is associated with the potential effects of aspartame consumption on neurodevelopmental processes, especially in children [16]. However, the data from controlled clinical trial results are contradictory. While some studies show that aspartame has no significant effect on ADHD symptoms, some studies indicate mild cognitive impairment [17]. In terms of effects on concentration and memory, aspartame is thought to affect neurotransmitter balance. Increased levels of phenylalanine and aspartic acid mainly affect excitatory neurotransmission via glutamate. This increases neurological excitability and leads to concentration problems in susceptible individuals [10].

The psychological effects of aspartame have been linked to potential effects on neurotransmitter levels. Phenylalanine, which is produced during the metabolism of aspartame, blocks the passage of tryptophan, the precursor of serotonin, into the brain. This leads to a decrease in serotonin levels, causing depression, anxiety, and mood swings [19].

Some studies have shown that a high‐dose aspartame consumption may increase depressive symptoms. For example, a 2014 study observed mood changes and increased depressive symptoms in individuals consuming aspartame at a daily dose of 25 mg/kg [20].

However, these effects are usually limited to high‐dose consumption and are not evident at normal dietary levels. Changes in neurotransmitter levels may be related to phenylalanine, aspartic acid, and methanol metabolism. These components may affect brain chemistry and contribute to anxiety, irritability, and memory problems [21].

#### 4.1.7. Clinical Studies Associated With Aspartame Consumption

Long‐term cohort studies examining aspartame’s effects on health provide important data in large populations. Such studies have been used to examine the association between aspartame consumption and chronic diseases such as obesity and diabetes, cardiovascular disease, and cancer [22]. For example, in a study conducted in the USA, no significant association was found between aspartame consumption and overall mortality in an analysis of more than 400,000 people. The same study reported that diet beverage consumption was associated with some metabolic risk factors, but this effect could not be directly attributed to aspartame [23].

Meta‐analyses and systematic reviews on aspartame combine the existing literature to provide more comprehensive results. A 2017 meta‐analysis evaluated 56 studies examining the effects of aspartame on cancer risk, metabolic syndrome, and neurological disorders. The results showed that aspartame is safe when consumed within ADI limits [24].

However, some reviews have indicated that aspartame may cause adverse effects in some people due to individual sensitivities and genetic factors. This indicates that the effects of aspartame may vary from person to person [25].

#### 4.1.8. Special Risk Groups

PKU is a rare disease caused by a genetic defect in phenylalanine metabolism. Phenylalanine, which is released as a result of the metabolism of aspartame, is toxic for PKU patients. In these individuals, phenylalanine accumulation leads to neurological damage, mental retardation, and behavioral disorders [26]. Therefore, patients with PRU should avoid aspartame products. Many countries around the world have made the labeling of aspartame‐containing products mandatory. The warning “Contains phenylalanine” is critical to inform PKU patients [27].

The effects of aspartame on children, pregnant women, and the elderly have been carefully studied due to different physiological characteristics. The safety of aspartame in children is important because of its potential effects on neurodevelopmental processes.

Studies have shown that aspartame consumption in children is safe; however, it is recommended to avoid excessive consumption [13]. Aspartame consumption during pregnancy is generally considered safe, but caution should be exercised for women who are carriers of PKU. Furthermore, the effects of aspartame differ in elderly carriers of PKU due to decreased metabolic rate and prevalence of chronic diseases [28].

## 5. Conclusion

Aspartame is an artificial sweetener with low calories and high sweetness intensity that is widely used in the food industry. In this review, we have tried to provide a comprehensive review of aspartame, from its chemical structure to its metabolism processes, potential health effects, and safety assessments. The discovery and industrial development of aspartame started with an accidental discovery during chemical research, especially in the 1960s, and soon became an important commercial product on a global scale. Regulatory agencies have conducted extensive safety assessments to ensure the safe use of aspartame in food products.

International authorities such as the US FDA and the EFSA have reported that aspartame is safe when consumed at ADI levels.

Studies on the chemical structure and metabolism of aspartame show that this sweetener is broken down in the body into components such as L‐aspartic acid, L‐phenylalanine, and methanol. These components are similar to substances found naturally in the body or ingested in the daily diet. The metabolic processes of aspartame start in the digestive tract and are completed in the liver. Phenylalanine and aspartic acid are involved in protein synthesis and neurotransmitter production, while methanol is rapidly metabolized in the body to formaldehyde and formic acid. These processes create different risk profiles, especially in individuals with certain genetic predispositions. The health effects of aspartame have been the subject of debate in the scientific community for many years. Research in areas such as cancer risk and neurological disorders, metabolic diseases, and cardiovascular effects has yielded conflicting findings. While some animal studies suggest that consumption of high doses of aspartame can lead to carcinogenic effects, these findings have not been consistently confirmed in human studies. Likewise, reports of neurological effects such as headache, depression, and seizures are explained by individual differences and dose‐dependent responses. In general, however, controlled clinical trials show that aspartame does not cause neurological disorders when consumed at recommended doses.

In terms of metabolic health, the effects of aspartame on obesity, insulin resistance, and glucose metabolism have also been emphasized. Aspartame’s low‐calorie nature offers advantages for weight management and diabetes control.

Some studies have raised concerns about potential effects on insulin response and the gut microbiota. However, these effects have generally been associated with high‐dose exposure, and daily dietary intakes are unlikely to lead to such consequences. Aspartame has a low glycemic index, making it a safe sweetener alternative for people with diabetes.

The effects of aspartame in special risk groups are significant for vulnerable populations such as PKU patients, children, pregnant women, and the elderly. Due to impaired phenylalanine metabolism in PKU patients, aspartame consumption can cause severe neurological problems. For this reason, products containing aspartame must carry the warning “contains phenylalanine.” As for the effects on children and pregnant women, available data suggest that aspartame is safe.

In June 2023, 25 people from 12 different countries formed a working group and initiated a study at IARC in Lyon, France, to determine whether aspartame and two other substances pose a particular cancer risk to human health. This study was that aspartame could pose a possible carcinogenic risk to human health, and IARC classified aspartame as a “Group 2B probable human carcinogen” [32]. Immediately following IARC’s cancer hazard identification meeting, the Joint FAO/WHO Expert Committee on Food Additives (JECFA) began a risk assessment study, including a review of the ADI of aspartame [33]. On July 14, 2023, in a joint press release, the International Agency for Research on Cancer (IARC), the Joint Expert Committee on Food Additives (JECFA), the WHO, and the Food and Agriculture Organization (FAO) issued a final assessment of the health effects of aspartame. Accordingly, with “limited evidence” for human cancer risk, IARC reaffirmed its classification of aspartame as possibly carcinogenic to humans (IARC Group 2B). JECFA reaffirmed the ADI of 40 mg/kg body weight [33].

Clinical trials, long‐term cohort studies, and meta‐analyses on the health effects of aspartame show that it is safe for the general population. However, individual differences, genetic predispositions, and overconsumption pose specific health risks. This makes it necessary to exercise caution in the consumption of aspartame, especially for vulnerable groups, where risk assessments should be carried out meticulously.

## Funding

No funding was received for this manuscript.

## Conflicts of Interest

The authors declare no conflicts of interest.

## Data Availability

This review does not generate any new dataset but synthesizes previously published data.
